# Supplementary material for: Multi-Wavelength Photobiomodulation Ameliorates Sodium Iodate-Induced Age-Related Macular Degeneration in Rats
Source: Int J Mol Sci. 2023 Dec 12;24(24):17394. doi: 10.3390/ijms242417394 (PMC10743884; doi:10.3390/ijms242417394)
Supplement: Supplementary file 1 [file ijms-24-17394-s001.zip › ijms-2682003-supplementary.pdf]

Supplementary file

Supplementary file S1: Figure of cell viability by power intensity for each single wavelength.

Supplementary file S2: Figure of quantification of intracellular ROS fluorescence intensity by PBM irradiation after hydrogen peroxide-induced oxidative stress.

Supplementary figures

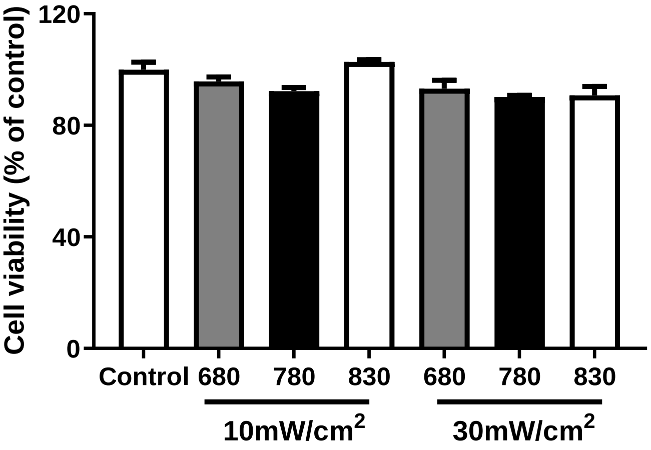

Figure S1. Single-wavelength PBM has no toxicity on the cell viability of ARPE-19 cells. Each individual wavelength also had no impact on cell viability, validating the conclusion that PBM does not exhibit toxicity to cells.

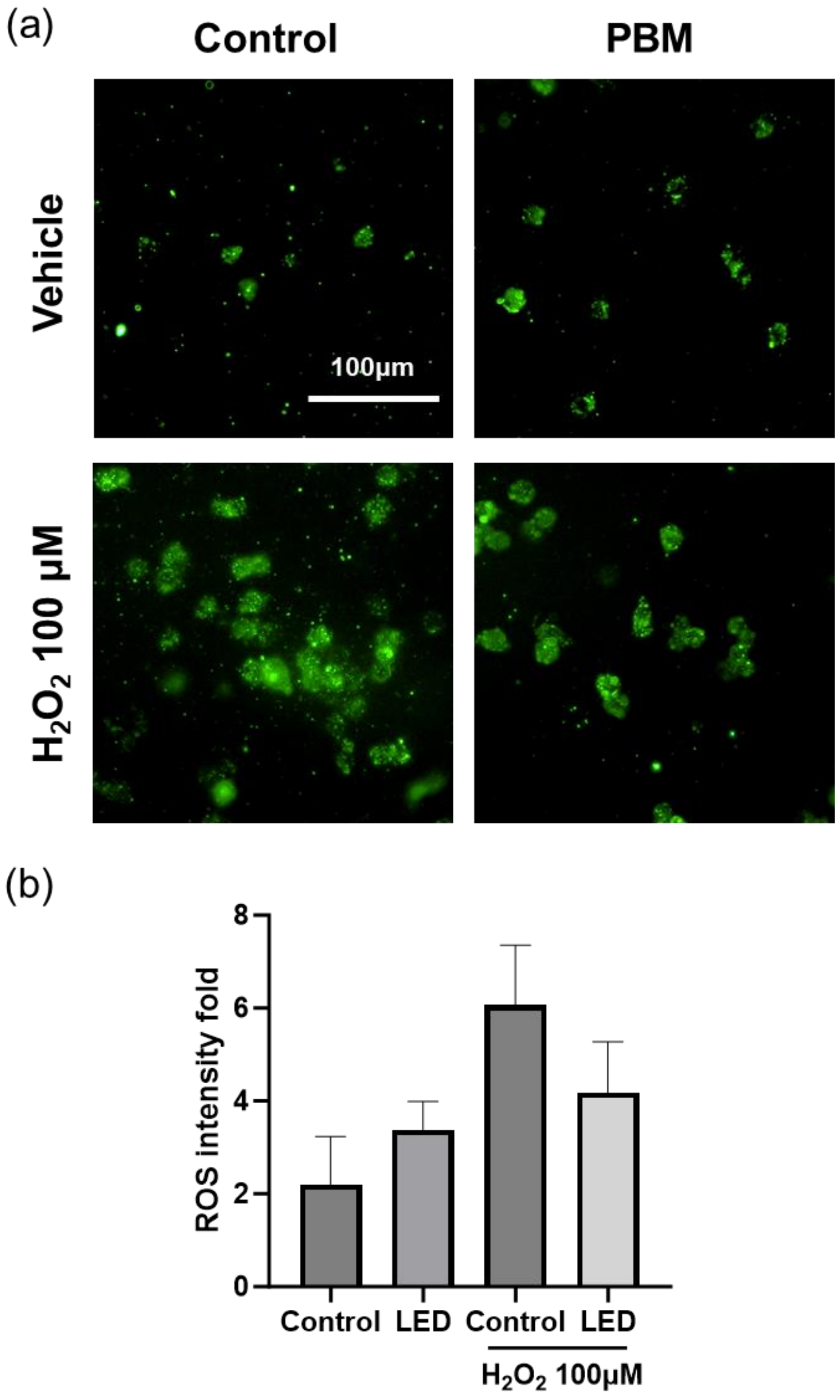

Figure S2. Quantification of intracellular ROS fluorescence intensity by PBM irradiation after hydrogen peroxide-induced oxidative stress. (a) Representative images of ROS (green) stained ARPE-19 cells after hydrogen peroxide-induced oxidative stress. Following the treatment of ARPE-19 cells with 100 μM hydrogen peroxide for 30 minutes to induce oxidative stress, we applied multi-wavelength PBM for 2 minutes to observe ROS changes. (b) The bar graph indicated ROS intensity fold after the treatment of hydrogen peroxide and multi-wavelength PBM. The results did not reach statistical significance.
